# Evaluating Ground State Energies of Chemical Systems with Low-Depth Quantum Circuits and High Accuracy

**DOI:** 10.1021/acs.jpca.4c07045

**Published:** 2025-03-03

**Authors:** Shuo Sun, Chandan Kumar, Kevin Shen, Elvira Shishenina, Christian B. Mendl

**Affiliations:** † School of Computation, Information and Technology, Technical University of Munich, Boltzmannstraße 3, Garching 85748, Germany; ‡ BMW Group Central Invention, Munich 80788, Germany; § applied Quantum algorithms (aQa), Leiden University, Leiden 2311 EZ, the Netherlands; ∥ Quantinuum, Leopoldstraße 180, Munich 80804, Germany; ⊥ Technical University of Munich, Institute for Advanced Study, Lichtenbergstraße 2a, Garching 85748, Germany; # Munich Center for Quantum Science and Technology (MCQST), Schellingstraße 4, Munich 80799, Germany

## Abstract

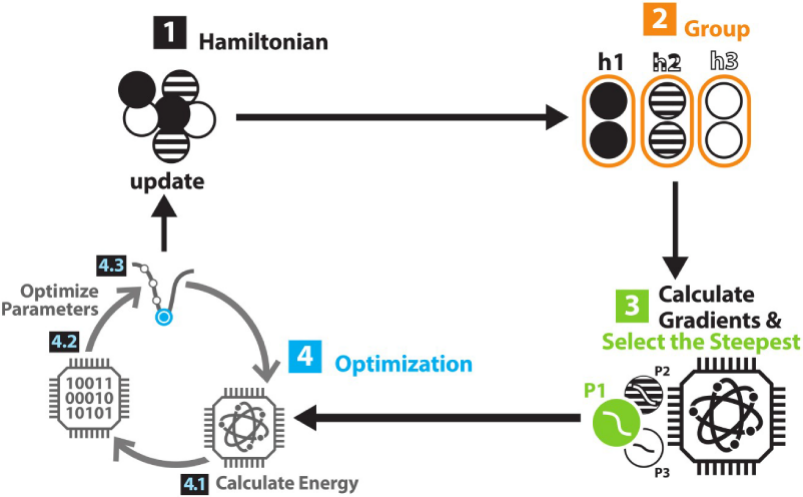

Quantum computers have the potential to efficiently solve the electronic structure problem but are currently limited by noise and shallow circuits. We present an enhanced Variational Quantum Eigensolver (VQE) ansatz based on the Qubit Coupled Cluster (QCC) approach that requires optimization of only *n* parameters, where *n* is the number of Pauli string generators, rather than the typical *n* + 2*m* parameters, where *m* is the number of qubits. We evaluate the ground state energies and molecular dissociation curves of strongly correlated molecules, namely O_3_ and Li_4_, using active spaces of varying sizes in conjunction with our enhanced QCC ansatz, Unitary Coupled Cluster Single–Double (UCCSD) ansatz, and the classical Coupled Cluster Singles and Doubles (CCSD) method. Compared to UCCSD, our approach significantly reduces the number of parameters while maintaining high accuracy. Numerical simulations demonstrate the effectiveness of our approach, and experiments on superconducting and trapped-ion quantum computers showcase its practicality on real hardware. By eliminating the need for symmetry-restoring gates and reducing the number of parameters, our enhanced QCC ansatz enables accurate quantum chemistry calculations on near-term quantum devices for strongly correlated systems.

## 1 Introduction

Solving electronic structure problems is the cornerstone of computational chemistry, enabling us to unravel the dynamic and kinetic properties of the chemical systems. In recent years, quantum computing has emerged as a promising avenue for efficiently simulating quantum systems. The Variational Quantum Eigensolver (VQE), which is first developed by Peruzzo et al.,^1^ stands out as a leading algorithm for near-term quantum devices due to the shallow circuit and noise resiliency.^2−5^ VQE exploits the variational nature of the ground state energy, i.e., , where  is the initial state, *U*(θ) is the parametrized unitary gate, which is often referred as “ansatz”, and *H* is the Hamiltonian. Under Born–Oppenheimer approximation, the electronic Hamiltonian of a molecular system is given by
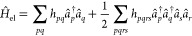
1in the second quantization form, where *p*, *q*, *r,* and *s* are spin orbitals. Various ansätze have been explored within VQE, such as Unitary Coupled Cluster (UCC),^6−10^ Qubit Coupled Cluster (QCC),^11−13^ the Hardware Efficient Ansatz (HEA),^14,15^ and the Adaptive Ansatz,^16−20^ each tailored to capture specific features of the electronic structure.

To achieve the global minimum, namely, the ground state energy, an optimization loop is essential. However, many aforementioned ansätze require a plethora of parameters, deepening the circuits and making the optimization process computationally intensive. Additionally, estimating the gradient often demands a significant number of circuit executions, further exacerbating the computational load.^21−23^ In this article, we address the challenges posed by the large number of parameters and deep circuits by an enhanced QCC approach. In contrast to the original QCC ansatz, our enhanced approach starts from a Hartree–Fock state, ensuring the correct particle number from the beginning. In this way, we eliminate the need for a series of single-qubit rotation gates in our enhanced approach to adjust the total particle number, as was the case in the original QCC ansatz. Then a sequence of Pauli string time evolution gates *e*^*-itP*^, where *P* is a tensor product of single-qubit Pauli operators, are applied. We highlight the work by Genin et al. on the application of iQCC for exploring the transition energies of phosphorescent complex inorganic systems.^24^ Their results demonstrated the high applicability of iQCC in applied materials research. In this work, we independently developed and implemented enhanced QCC, and conducted experiments both with a classical simulator and with two real quantum hardware. With the enhanced QCC algorithm, we can obtain the ground state energies and molecular dissociation curves of two molecules: O_3_, which exhibits significant electron correlation,^25−27^ and Li_4_, which has attracted considerable attention in the fields of ultracold molecules and theoretical chemistry.^28−31^ This distinguishes them from simpler systems, such as hydrogen chains or LiH. Therefore, they provide valuable insights and present challenges for investigation. We conduct a comparative analysis of the performance and the number of parameters required by the enhanced QCC and UCCSD ansatz. Furthermore, we execute the parametrized QCC circuits on two distinct quantum computers and achieve near-chemical precision on one of the machines, which highlights the practical utility of our approach.^14,32,33^

For ease of notation, we follow the notation from Helgaker et al.^34^ in this article, where occupied orbitals are denoted as *ab*, virtual (unoccupied) orbitals as *mn*, inactive orbitals as *ijkl*, active orbitals as *uvxy*, and general orbitals as *pqrs*. We decorate operators with ' ^' to represent Fermionic operators; the ones without decoration are qubit operators.

## 2 Methods

### 2.1 Unitary Coupled Cluster

The inspiration for UCC ansatz stems from the Coupled Cluster (CC) theory in computational chemistry.^6^ The latter approach involves applying cluster operators to a reference state, typically the Hartree–Fock (HF) state, resulting in a linear combination of Slater determinants with various excitations. First, let us define the excitation operators *T̂*:^10^
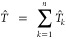
2a
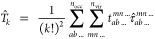
2b
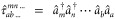
2c

In the case where only single and double excitations are considered, the excitation operator simplifies to
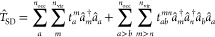
3

For the canonical CCSD method, the cluster operator is given by . However, this operator is not unitary in general and cannot be directly implemented on a quantum computer. Recognizing that *T̂* – *T̂*^†^ is a skew-hermitian operator, we instead apply  as the unitary cluster operator, which is suitable for quantum computers.

Since all the aforementioned operators are Fermionic, Fermion-to-qubit mappings such as Jordan–Wigner,^35^ Bravi–Kitaev,^36^ Parity mapping,^37^ etc. are required to transform them into qubit operators. In the context of UCCSD, the resultant qubit operators often turn out to be too intricate for direct implementation on quantum computers. Consequently, an additional decomposition is essential, leading to more complex circuits in practical applications.^38^

### 2.2 Qubit Coupled Cluster

The original QCC ansatz is both inspired by chemistry and designed for hardware efficiency. This approach uses a sequence of multiqubit Pauli string time evolution gates defined as
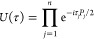
4where τ_*j*_ represents the coefficient, and *P*_*j*_ is the multiqubit Pauli string operator. These gates act on an unentangled, qubit-mean-field (QMF) state,^11,12^ which is expressed as a tensor product of qubit states with each qubit state defined as
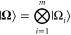
5
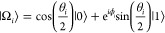
6

The QCC energy,
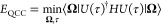
7is then minimized over the parameters θs, ϕs, and τs. Let us write the molecular Hamiltonian as sum of Pauli string operators
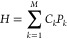
8

As we know that the electronic Hamiltonian is real when no external electric field is present,^39^ the energy gradient with respect to τ_*j*_ can be simplified to (see ref (12) for details):
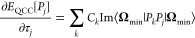
9where |**Ω**_**min**_⟩ is the QMF state with the lowest energy, i.e., |**Ω**_**min**_⟩ = arg min ⟨**Ω|***H***|Ω**⟩. With the assumption that the QMF state |**Ω**_**min**_⟩ is an eigenstate of all  operators, we can further reduce the computational cost by grouping the Pauli terms in the Hamiltonian that share the same flip index *F*(*P*) together to *h*_*n*_s, where
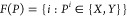
10

Only *P*_*j*_s that obsess the same flip index as the corresponding Hamiltonian subgroup *h*_*j*_ have nonzero contributions to the gradients, and they exhibit gradients with the same absolute value.

After selecting the Pauli strings *P*_*j*_s with the highest gradients, and optimizing the corresponding amplitudes τ_*i*_, we proceed to the next iteration. The Hamiltonian is updated by
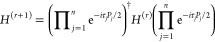
11

Here *n* is the number of Pauli strings that are applied in each iteration, and *r* is the iteration index.

One caveat of QCC is that the parameters for the initial state scale linearly with the system size, and optimization challenges, such as the Barren plateaus,^40,41^ can arise.

In this work, we aim to develop an ansatz that uses low-depth circuits and fewer parameters. A key observation driving our approach is the nonconservation of the particle number inherent in arbitrary Pauli time evolution gates, i.e., . However, in the context of a chemical system, the ground state inherently possesses a well-defined particle number. Moreover, in a chemical system with a stable configuration, a state with a different particle number incurs a higher energy than a state with the correct particle number. To address this, the original ansatz introduces additional single-qubit rotation gates at its onset, as shown in eq 6, to guide the state to the ground state particle number sector.^42^

Our innovation comes from using a state with the correct particle number, like the HF state, as the initial state, followed by applying a series of Pauli string time evolution gates e^–*iτP*/2^. In this way, we can eliminate the need for single-qubit rotation gates. Additionally, it is noteworthy that in the molecular orbital basis, the HF state satisfies the assumption made in the original ansatz: it is indeed an eigenstate of all  operators. As a result, we can inherit the remainder of the original QCC ansatz.

**Figure 1:**
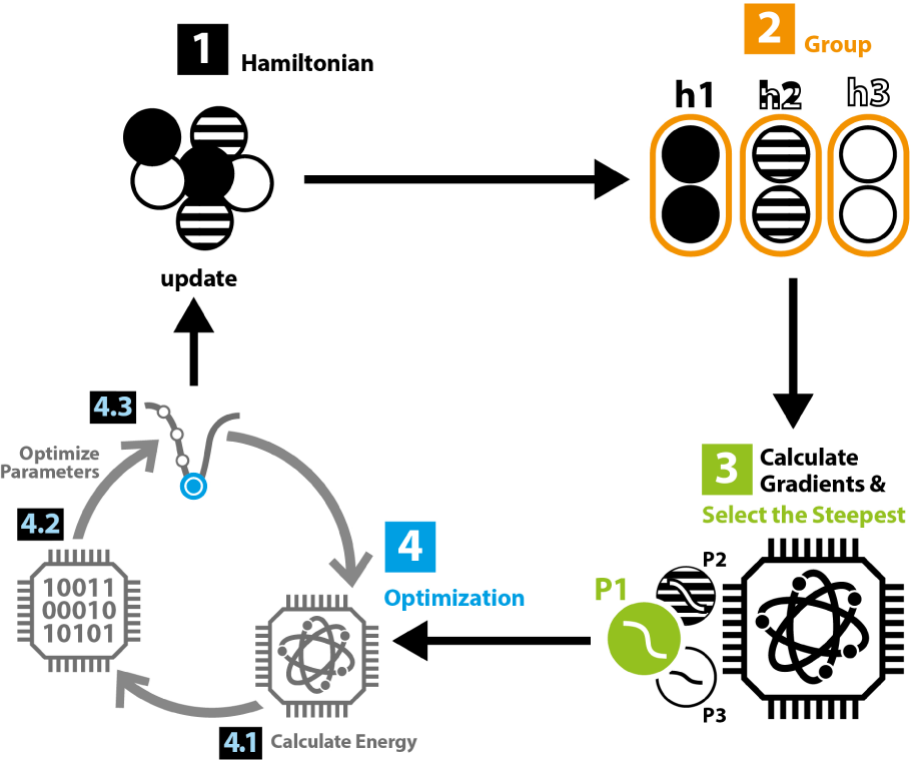
Schematic depiction of the VQE-enhanced QCC optimization loop. Step 2 and step 4.1 can be executed on quantum hardware. And other steps are done on classical computer. The initial state of step 2 and step 4.1 is Hartree–Fock state, which is not explicitly depicted here.

For a problem with *m* system qubits, preparing a QMF state, as defined in eq 6, requires 2*m* single-qubit rotation gates and parameters, along with *n* Pauli string time evolution gates for evolving the system toward gradient descent. In contrast, our enhanced approach allows the reduction of single-qubit rotation gates, resulting in a significant decrease in both the number of parameters and computational demand. The detailed algorithmic procedure is presented in Algorithm 1. And a schematic representation of the algorithm is illustrated in Figure 1.
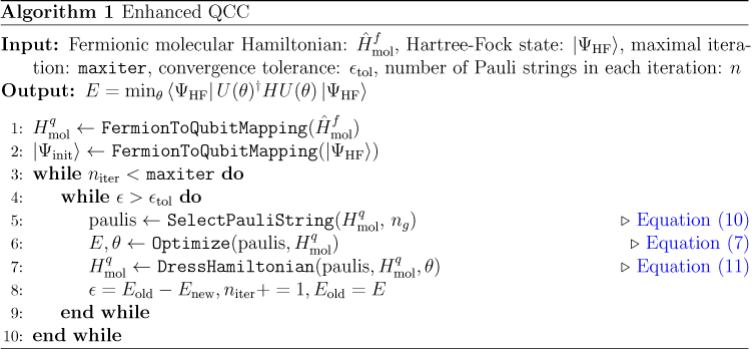


### 2.3 Complete Active Space

The Complete Active Space (CAS) approach is a powerful tool in computational chemistry, reducing the problem size while maintaining promising accuracy for the total energy calculation. Constrained by the available computing resources, we use CAS to reduce the problem size in the examples presented in Section 3.

The active space Hamiltonian, denoted as *Ĥ*_active_, is expressed as^43^
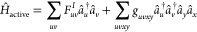
12where  is defined as
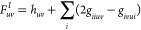
13and the inactive space energy is
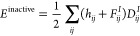
14

The one-body term in the Hamiltonian is updated to describe not only the kinetic energy of the active space electrons but also the energy contributed by the inactive electrons. The two-body term is defined as slices of the original two-body interaction. The total energy (*E*^total^) is then expressed as the sum of the active space energy (*E*^active^), the inactive space energy (*E*^inactive^), and the energy contributed by the nuclei (*E*^nuclei^):
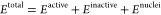
15

In this paper, we will use CAS(*e,o*) to represent the active space size, where *e* is the number of electrons, and *o* is the number of spatial orbitals.

## 3 Results and Discussions

In this section, we numerically and experimentally demonstrate our algorithm by computing the ground state energies and the potential energy surfaces of the O_3_ and Li_4_ molecules with active space sizes (2,2), (4,4), and (6,6). All calculations utilize the cc-pVDZ basis set^44^ and the Parity mapping.^37^ The active space Hamiltonians, CCSD, and CASCI results are obtained using PySCF.^45^ Quantum circuit simulations are carried out using the noiseless statevector simulator in Qiskit,^46^ and energy minimization is performed with the L-BFGS-B method implemented in SciPy.^47^ Experimental results obtained from runs on quantum hardware are detailed in Section 3.4. Additionally, molecular coordinates, electron numbers, and spin numbers obtained from CCSD and QCC calculations are provided in the Supporting Information.

### 3.1 Convergence

**Figure 2:**
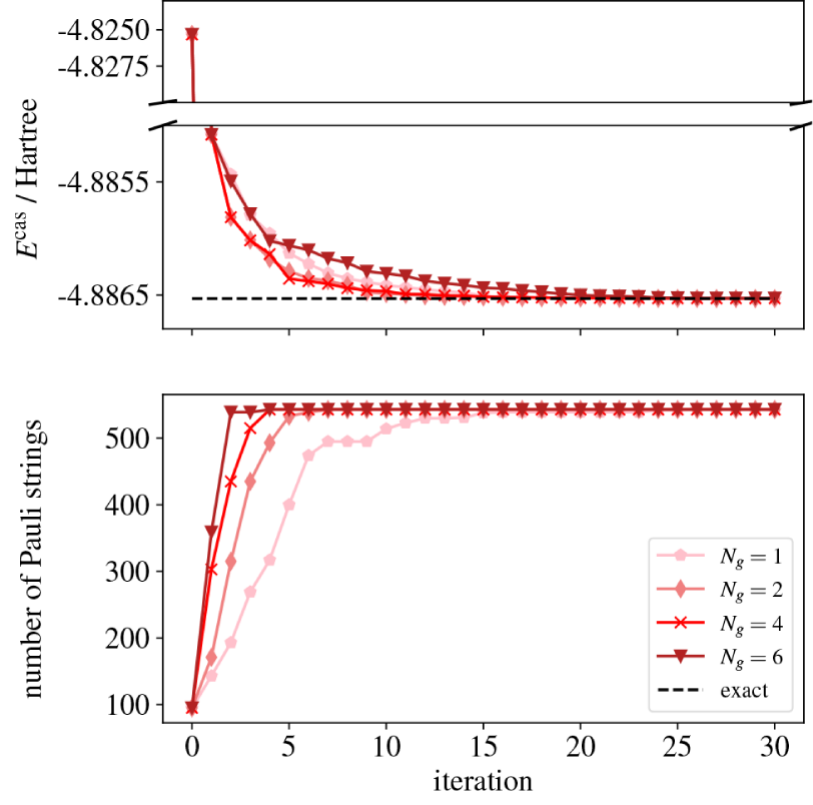
Energy convergence (upper panel) and number of terms in the Hamiltonian (lower panel) using enhanced QCC ansatz with different numbers of generators for O_3_ at *d* = 1.28 Å.

We first focused on examining the convergence behavior of our algorithm, particularly in the context of ground state energy estimation for O_3_ and Li_4_ in two configurations. The representative result, illustrated in Figure 2, revealed interesting patterns as we varied the number of generators. Notably, we found that altering the number of generators at each iteration did not have a significant impact on the convergence energy or the number of terms in the final Hamiltonian. This observation suggested the robustness of our algorithm, regardless of the specific generator count.

Moreover, as depicted in Figure 2, an increase in the number of generators at each iteration exhibited a tendency to accelerate convergence to some degree. Nevertheless, a critical threshold was observed beyond which the convergence rate diminished. This phenomenon is likely attributed to the exponential expansion of the search space with the growing number of generators, presenting a challenge for the optimizer to efficiently locate the optimal solution within a limited number of iterations.

Additionally, our investigation revealed that while the number of terms in the Hamiltonian saturated more rapidly with a higher number of generators, this saturation did not necessarily correlate with the convergence of energy, as shown in Figure 2. For the sake of optimization simplicity, we opted to use only one generator per iteration in the subsequent examples.

### 3.2 Potential Energy Surfaces

**Figure 3:**
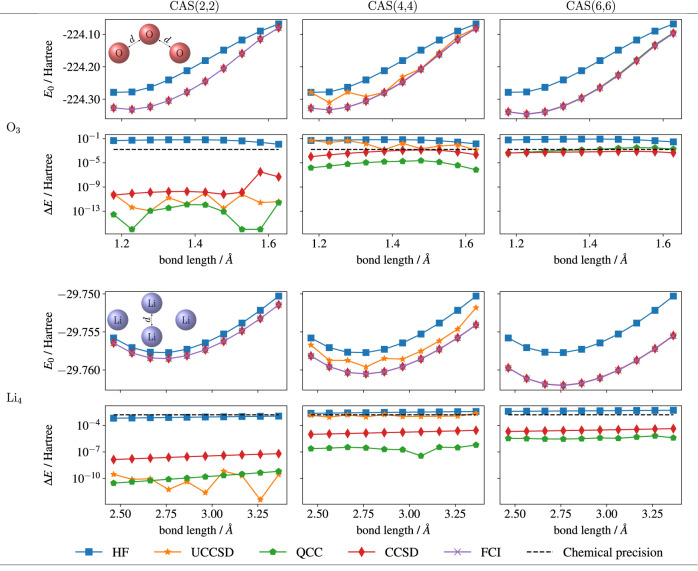
Potential energy surfaces of O_3_ and Li_4_, using CAS(2,2), (4,4), and (6,6) in conjunction with the enhanced QCC ansatz, UCCSD (Unitary Coupled Cluster Single–Double) ansatz, CCSD, and FCI as the active space solver. The upper panel of each figure describes the ground-state energies at different configurations. The bottom panel of each figure shows the energy difference between the chosen active space solver and CASCI (CAS with FCI as active space solver). For active space (6,6), due to the high parameter counts of the UCCSD method, we did not perform the PES calculation.

We investigated potential energy surfaces for various molecules using CAS(2,2), CAS(4,4), and CAS(6,6) with four distinct active space solvers: UCCSD, QCC, CCSD (coupled cluster single and double), and FCI (full configuration interaction). The results are presented in Figure 3.

First, we assessed the potential energy surface with an active space of (2,2), i.e., two electrons in two spatial orbitals. In this case, all the active space solvers achieved chemical precision, which we define as  Hartree, across all bond lengths for O_3_ and Li_4_.

However, when the active space was extended to (4,4), the UCCSD solver faced challenges. It failed to produce energies within chemical precision and even returned energies at the Hartree–Fock level for O_3_ at specific bond lengths (e.g., *d* = 1.18 and 1.28 Å). In contrast, the enhanced QCC solver consistently provided favorable results, occasionally surpassing the performance of the CCSD solver. This could arise from the inclusion of higher-order excitations in QCC, compared with the sole consideration of single and double excitations in CCSD.

**Figure 4:**
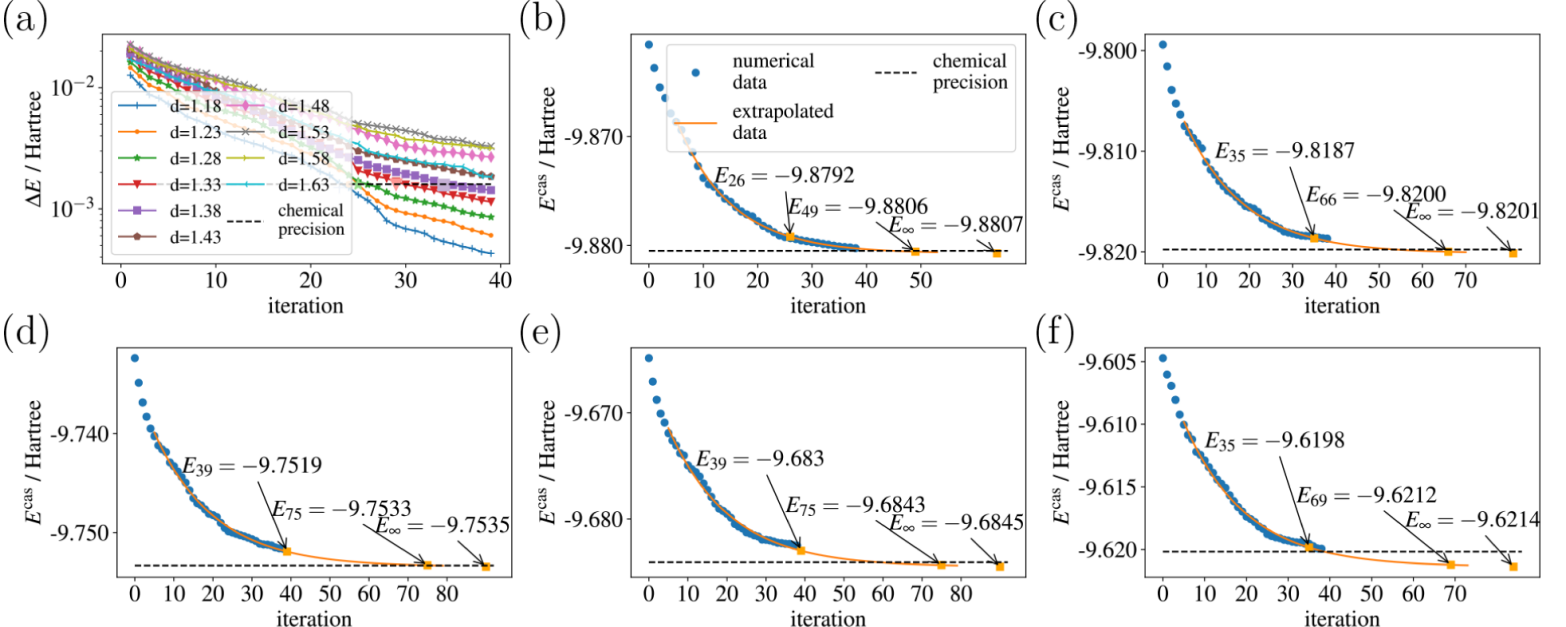
(a) Energy convergence curve of O_3_ with active space size (6,6). (b)–(f) are the extrapolated energy convergence curves at (b) *d* = 1.43, (c) *d* = 1.48, (d) *d* = 1.53, (e) *d* = 1.58, and (f) *d* = 1.63 Å. The first highlighted point of each figure corresponds to the iteration and energy by setting the value of *E*^(*i*)^ - *E*^(*i*+1)^ from eq 16b to 1.6 × 10^–3^ Hartree. The second highlighted point gives the iteration number and the expected energy when setting the value of *E*^(*i*)^ - *E*^(*i*+1)^ = 1.6 × 10^–4^ Hartree. The last highlighted point represents the expected energy with infinite iterations from the extrapolation.


For CAS(6,6), we refrained from optimizing the UCCSD ansatz due to its high parameter counts (as specified in Section 3.3). QCC, on the other hand, exhibited accurate results under chemical precision for Li_4_. However, occasional convergence issues were observed for O_3_ after 40 iterations. To address these challenges, we implemented an extrapolation method as suggested in ref (12). The energy difference exhibits exponential decay, as illustrated in Figure 4a. Consequently, a logarithmic relationship is assumed:

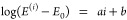
16a
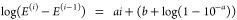
16bwhere the parameters *a* and *b* are obtained through fitting. The estimated value  is obtained by solving this fitting problem. The first five iterations were discarded, and the subsequent 35 iterations were employed to address the fitting problem. The results are presented in Figure 4. For *d* = 1.43–1.63 Å, all estimated energies fall within chemical precision. By imposing the condition *E*^(*i*)^ – *E*_exact_ < 1.6 × 10^–4^ Hartree and identifying the necessary iteration *i*, the energy within chemical precision is successfully achieved as well.

These findings support the idea that under the condition that available resources are limited, it may not be necessary to execute the QCC ansatz until the energy difference is smaller than a specific threshold or reaches the maximum iteration limit. Instead, it is already sufficient to run the optimization for a few iterations and subsequently perform extrapolation.

### 3.3 Parameter Number Count

The determination of parameter numbers in the UCCSD ansatz follows a straightforward pattern. For an active space of size (2,2), there are 2 single excitations and 1 double excitation, so 3 parameters are needed. Expanding the active space to (4,4) results in 8 single excitations and 18 double excitations, which means a total of 26 parameters. In the case of an active space with a size of (6,6), the count increases to 18 types of single excitations and 99 double excitations, leading to 117 parameters. Consequently, as the active space size increases, classical optimization becomes exponentially more challenging.

In contrast, the enhanced QCC ansatz, as illustrated in Table 1, exhibits notable efficiency. Specifically, for an active space size of (2,2), the energies of O_3_ and Li_4_ fall below chemical precision after just one iteration, equivalent to one Pauli string time evolution gate. With an active size of (4,4), the high dimensionality of the UCCSD ansatz occasionally hampers the optimizer from finding the ground state, as is evident in Figure 3. However, with the enhanced QCC ansatz, chemical precision is achieved within a maximum of 4 layers of Pauli string time evolution gates, and for each iteration, only one parameter requires optimization. This stark contrast underscores the efficiency and computational advantages of the enhanced QCC ansatz over the UCCSD ansatz in quantum chemistry calculations.

**Table 1:** Number of Optimization Parameters

	CAS(2,2)		CAS(4,4)		CAS(6,6)	
	O_3_	Li_4_	O_3_	Li_4_	O_3_	Li_4_
QCC	1	1	1–4	1–2	24–75	3
UCCSD	3	3	26	26	117	117

### 3.4 Experimental Results on Quantum Hardware

Our experiments were conducted on two distinct quantum hardware platforms: the superconducting-based IBM Kolkata quantum computer^48^ and the trapped-ion-based Quantinuum H1-1 quantum computer.^49^ The hardware parameters for both devices are outlined in Table 2. Due to constraints such as the number of jobs we can submit and the total runtime available, we chose to run the circuit using classically optimized parameters instead of conducting the entire VQE optimization loop on the quantum computers. The shot number was consistently set to 10^4^ for both devices during the experimental runs.

**Table 2:** Hardware Parameters

	qubit type	single-qubit gate infidelity	two-qubit gate infidelity
IBM Kolkata	superconducting qubits	2.199e-4	7.743e-3
Quantinuum H1-1	trapped-ion qubits	4e-5	2e-3

**Figure 5:**
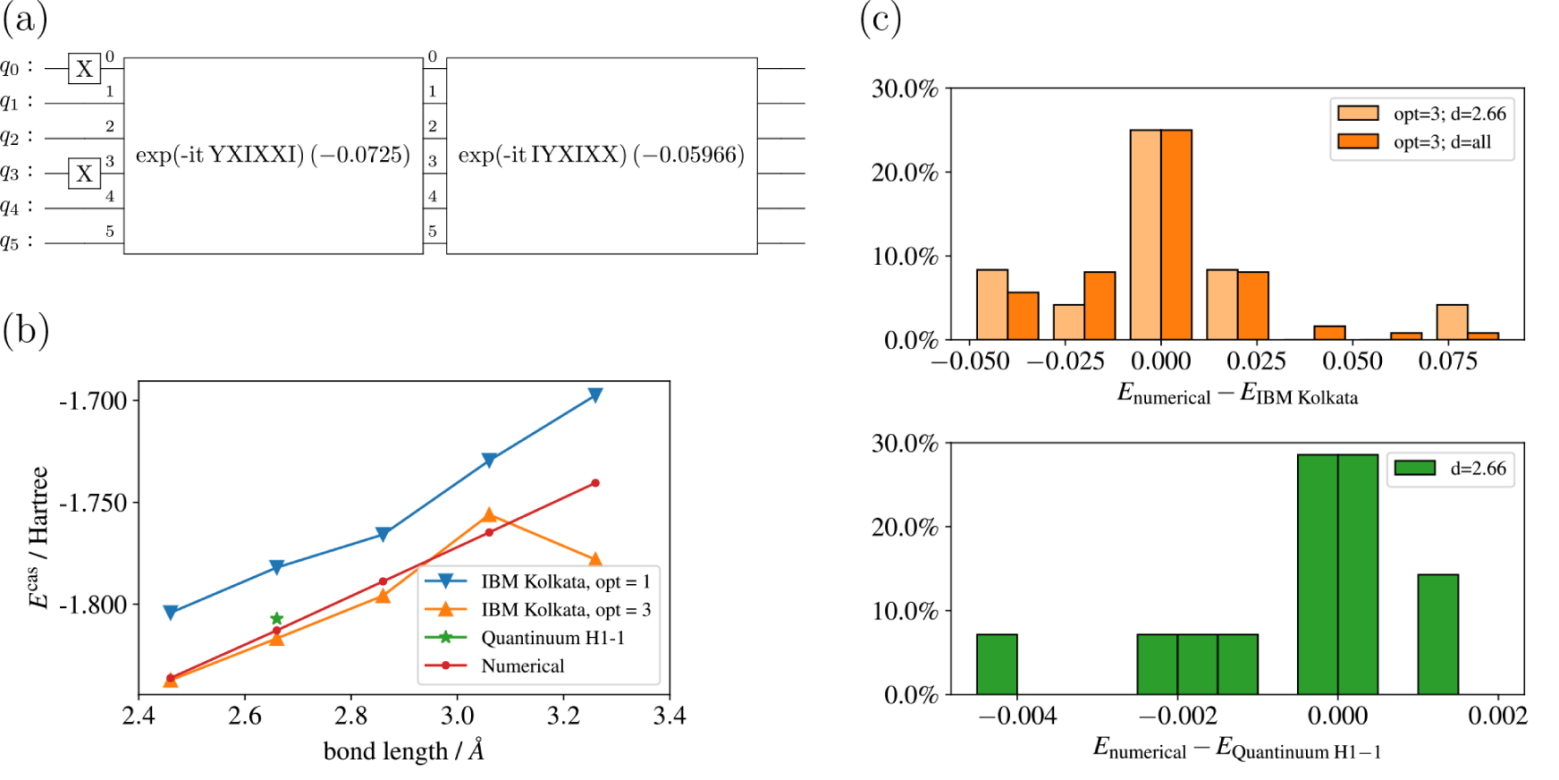
(a) is the quantum circuit for obtaining the active space energy of Li_4_. (b) shows the active space energies with different bond length of Li_4_ from IBM Kolkata and Quantinuum H1-1 quantum computer. (c) shows the distribution of the energy difference between the numerical energies *E*_numerical_ and the measured energy *E*_experimental_ for each commuting group of the Li_4_ Hamiltonian on IBM Kolkata machine and Quantinuum H1-1 machine.

We took Li_4_ with CAS (4,4) as a representative example. Due to constraints on the coherence time of the qubits, we limited our application to the first two layers of Pauli string time evolution gates, as illustrated in Figure 5a, because the numerical study in the last section has shown that two layers of Pauli string time evolution gates are sufficient to lower the energy to the chemical precision level. To improve the efficiency of the measurement process, we grouped qubit-wise commuting Pauli strings in the Hamiltonian, allowing for simultaneous measurements.^50,51^

We performed two sets of experiments on the IBM Kolkata platform, one with the optimization level set to 1, and the other set to 3.^52^ In each set, we measured the active space energy for the bond length ranging from 2.46 to 3.26 Å. The corresponding energy is plotted in Figure 5. With the optimization level set to 1, the energy trend aligns with the numerically simulated energy, albeit with an offset of approximately 0.4 Hartree, which is two magnitudes higher than the chemical precision. Conversely, with the optimization level set to 3, the discrepancy between the experimental and numerical results diminished across most bond lengths, leaving an offset in the order of 10^–2^ Hartree.

On Quantinuum H1-1 quantum computer, we assessed the active space energy of Li_4_ at bond length of 2.66 Å with optimization level set to 2.^53,54^ The total energy deviation *E*_num_ – *E*_exp_ is 0.0067 Hartree.

Additionally, the energy difference between numerically simulated and experimentally measured energies for each commuting group under the highest optimization level of both the IBM Kolkata machine and the Quantinuum H1-1 machine is displayed in Figure 5. The errors within each commuting group fluctuate within the range of −0.05 to 0.08 Hartree for IBM Kolkata and −0.005 to 0.002 Hartree for Quantinuum H1-1. In many cases, although not universally, these errors tend to cancel each other out. This observation suggests that by measuring the same circuit a sufficiently large number of times on a NISQ device, we can obtain results with a decent level of accuracy.

## 4 Conclusion

In this study, we introduced a Variational Quantum Eigensolver ansatz based on the Qubit Coupled Cluster ansatz. We assessed its performance through both numerical simulations and experimental trials on quantum computers. Numerical simulations showcased the efficiency of our enhanced QCC ansatz, revealing a balance of low circuit depth and parameter counts and high accuracy in handling both weakly correlated and strongly correlated systems. Experimental results obtained from quantum hardware experiments demonstrated that despite inherent noise and other hardware limitations, our proposed ansatz achieved energy measurements at a near-chemical precision level. This outcome underscored the robustness and practicality of our approach in real-world quantum computing settings.

In contrast to the original QCC ansatz, which utilized Pauli string time evolution gates that are in general not particle number conserving for energy gradient descent and single-qubit rotation gates for symmetry restoration, our modified approach eliminated the need for single-qubit rotation gates. This was achieved by initiating and maintaining computations within the Hilbert space section with the correct symmetry. Consequently, this reduction in gate layers and required parameters not only accelerates the computational process but also raises intriguing questions. On the one hand, the symmetry-breaking and restoring approach may yield shortcuts, accelerating the search for the ground state and ground state energy. On the other hand, it extends the problem space, introducing the possibility of multiple optimal solution paths for a given problem. This opens up topics for further research, especially in the comparative analysis of symmetry-breaking and restoring methods against symmetry-conserving methods. Future investigations in this direction will deepen our understanding and contribute to the refinement of quantum algorithms for chemical systems.

## Data Availability

The Pauli gates, their coefficients, and the demonstration of this work are available at https://github.com/sunshuo987/qcc_demo_data. Additionally, molecular coordinates, electron numbers, and spin numbers are provided in the Supporting Information.

## Supporting Information Available

Molecular coordinates; energy convergence with different optimizers; occupation and spin numbers (PDF)
