# Supplementary material for: Evaluating Ground State Energies of Chemical Systems with Low-Depth Quantum Circuits and High Accuracy
Source: J Phys Chem A. 2025 Mar 3;129(10):2379–86. doi: 10.1021/acs.jpca.4c07045 (PMC11912482; doi:10.1021/acs.jpca.4c07045)
Supplement: Supplementary file 1 — jp4c07045_si_001.pdf [file jp4c07045_si_001.pdf]

Supporting Information: Evaluating Ground State Energies of Chemical Systems with Low-Depth Quantum Circuits and High Accuracy

Shuo Sun[*,†] Chandan Kumar[‡] Kevin Shen[‡,¶] Elvira Shishenina[§] and Christian B. Mendl[*,†,‖,⊥]

†*Technical University of Munich, School of Computation, Information and Technology, Boltzmannstraße 3, 85748 Garching, Germany*

‡*BMW Group Central Invention, 80788 Munich, Germany*

¶*applied Quantum algorithms (aQa), Leiden University, The Netherlands*

§*Quantinuum, Leopoldstraße 180, 80804 Munich, Germany*

‖*Technical University of Munich, Institute for Advanced Study, Lichtenbergstraße 2a, 85748 Garching, Germany*

⊥*Munich Center for Quantum Science and Technology (MCQST), Schellingstraße 4, 80799 Munich, Germany*

E-mail: shuo.sun@tum.de; christian.mendl@tum.de

1 Molecular coordinates

Table S1: Molecular coordinates of O_3. In this work, l ranges from -0.1 Å to 0.4 Å.

Atom	X (Å)	Y (Å)	Z (Å)
O	0.0000	0.0000	0.0000
O	0.0000	$(1.278 + l) \cdot \sin(116.8°)$	$(1.278 + l) \cdot \cos(116.8°)$
O	0.0000	$-(1.278 + l) \cdot \sin(116.8°)$	$(1.278 + l) \cdot \cos(116.8°)$

Table S2: Molecular coordinates of Li_4. In this work, l ranges from -0.1 Å to 0.4 Å.

Atom	X (Å)	Y (Å)	Z (Å)
Li	-2.7074	0.0000	0.0000
Li	0.0000	-(1.3317+l)	0.0000
Li	0.0000	1.3317+l	0.0000
Li	2.7074	0.0000	0.0000

2 Energy convergence with different optimizers

In addition to the L-BFGS-B optimization method, we investigated two alternative optimization algorithms: the Nelder-Mead and Powell optimizers. As shown in Figure S1, while both optimizers ultimately converged to the same energy value, the Powell optimizer demonstrated faster convergence compared to the Nelder-Mead optimizer and exhibited similar convergence performance to the L-BFGS-B optimizer.

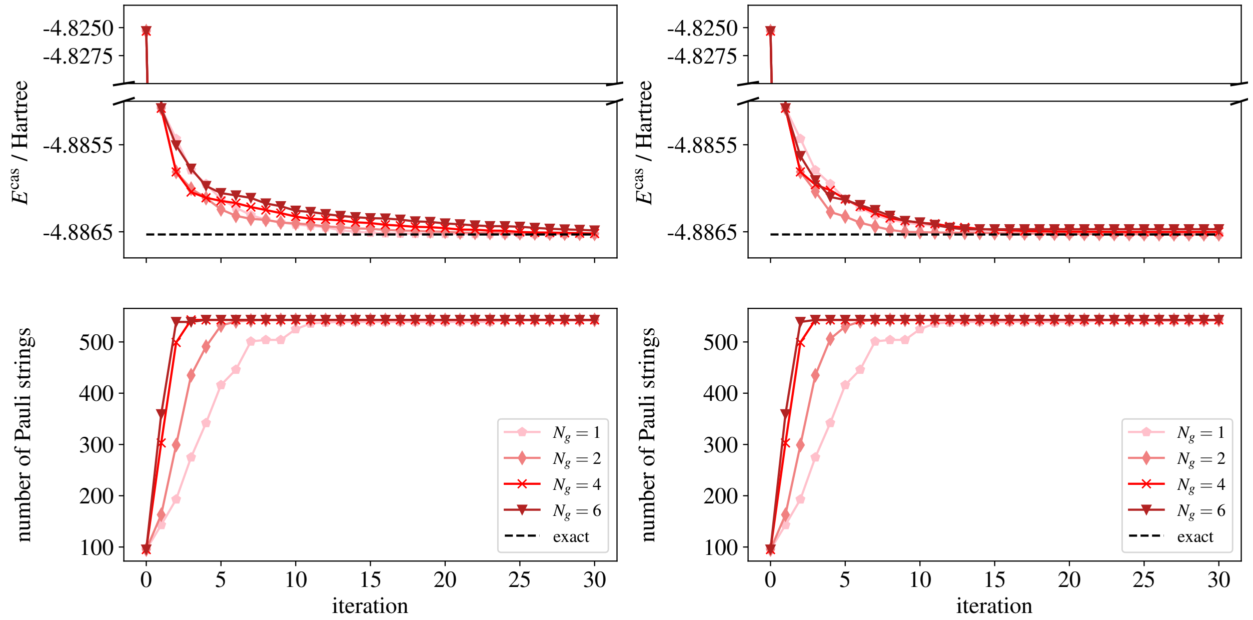

(a) Nelder-Mead Optimization

(b) Powell Optimization

Figure S1: Comparison of optimization methods: (a) Nelder-Mead and (b) Powell. The upper panels illustrate energy convergence, while the lower panels show the number of terms in the Hamiltonian. These results are obtained using the enhanced QCC ansatz with varying numbers of generators for O_3 at $d = 1.28$ Å.

3 Occupation and spin numbers

We calculated the electron occupation numbers for the highest occupied molecular orbital (HOMO) and lowest unoccupied molecular orbital (LUMO) for the states obtained using the CCSD and QCC methods. These values are denoted as $Ne^{\text{orbital}}_{\text{method}}$. The difference in electron numbers is defined as $\Delta Ne = Ne_{\text{CCSD}} - Ne_{\text{QCC}}$.

In addition, we computed the spin-related quantum numbers S_z and S^2 for the corresponding states. The detailed data is presented in Table S3 through Table S8.

Table S3: Orbital occupation number and spin numbers of O_3 with CAS(2,2)-CCSD/QCC.

bond length	$Ne_{\text{CCSD}}^{\text{HOMO}}$	$Ne_{\text{QCC}}^{\text{HOMO}}$	ΔNe^{HOMO}	$Ne_{\text{CCSD}}^{\text{LUMO}}$	$Ne_{\text{QCC}}^{\text{LUMO}}$	ΔNe^{LUMO}	$Ne_{\text{QCC}}^{\text{Total}}$	S_z	S^2
1.18	1.8302	1.8302	0.0000	0.1698	0.1698	0.0000	2.0000	0.000000	0.000000
1.23	1.7974	1.7974	0.0000	0.2026	0.2026	0.0000	2.0000	0.000000	0.000000
1.28	1.7667	1.7667	0.0000	0.2333	0.2333	0.0000	2.0000	0.000000	0.000000
1.33	1.7419	1.7419	0.0000	0.2581	0.2581	0.0000	2.0000	0.000000	0.000000
1.38	1.7279	1.7279	0.0000	0.2721	0.2721	0.0000	2.0000	0.000000	0.000000
1.43	1.7320	1.7320	0.0000	0.2680	0.2680	0.0000	2.0000	0.000000	0.000000
1.48	1.7641	1.7641	0.0000	0.2359	0.2359	0.0000	2.0000	0.000000	0.000000
1.53	1.8310	1.8310	0.0000	0.1690	0.1690	0.0000	2.0000	0.000000	0.000000
1.58	1.9110	1.9110	0.0000	0.0890	0.0890	0.0000	2.0000	0.000000	0.000000
1.63	1.9626	1.9626	0.0000	0.0374	0.0374	0.0000	2.0000	0.000000	0.000000

Table S4: Orbital occupation number and spin numbers of Li_4 with CAS(2,2)-CCSD/QCC.

bond length	$Ne_{\text{CCSD}}^{\text{HOMO}}$	$Ne_{\text{QCC}}^{\text{HOMO}}$	ΔNe^{HOMO}	$Ne_{\text{CCSD}}^{\text{LUMO}}$	$Ne_{\text{QCC}}^{\text{LUMO}}$	ΔNe^{LUMO}	$Ne_{\text{QCC}}^{\text{Total}}$	S_z	S^2
2.46	1.9902	1.9902	0.0000	0.0098	0.0098	0.0000	2.0000	0.000000	0.000000
2.56	1.9891	1.9891	0.0000	0.0109	0.0109	0.0000	2.0000	0.000000	0.000000
2.66	1.9879	1.9879	0.0000	0.0121	0.0121	0.0000	2.0000	0.000000	0.000000
2.76	1.9866	1.9866	0.0000	0.0134	0.0134	0.0000	2.0000	0.000000	0.000000
2.86	1.9852	1.9852	0.0000	0.0148	0.0148	0.0000	2.0000	0.000000	0.000000
2.96	1.9836	1.9836	0.0000	0.0164	0.0164	0.0000	2.0000	0.000000	0.000000
3.06	1.9818	1.9818	0.0000	0.0182	0.0182	0.0000	2.0000	0.000000	0.000000
3.16	1.9799	1.9799	0.0000	0.0201	0.0201	0.0000	2.0000	0.000000	0.000000
3.26	1.9778	1.9778	0.0000	0.0222	0.0222	0.0000	2.0000	0.000000	0.000000
3.36	1.9754	1.9754	0.0000	0.0246	0.0246	0.0000	2.0000	0.000000	0.000000

Table S5: Orbital occupation number and spin numbers of O_3 with CAS(4,4)-CCSD/QCC.

bond length	$Ne^{\mathrm{HOMO}}_{\mathrm{CCSD}}$	$Ne^{\mathrm{HOMO}}_{\mathrm{QCC}}$	$\Delta Ne^{\mathrm{HOMO}}$	$Ne^{\mathrm{LUMO}}_{\mathrm{CCSD}}$	$Ne^{\mathrm{LUMO}}_{\mathrm{QCC}}$	$\Delta Ne^{\mathrm{LUMO}}$	$Ne^{\mathrm{Total}}_{\mathrm{QCC}}$	S_z	S^2
1.18	3.8288	3.8272	0.0016	0.1712	0.1728	-0.0016	4.0000	0.000000	0.000000
1.23	3.7962	3.7940	0.0022	0.2038	0.2060	-0.0022	4.0000	0.000000	0.000000
1.28	3.7655	3.7626	0.0029	0.2345	0.2374	-0.0029	4.0000	0.000000	0.000000
1.33	3.7405	3.7365	0.0040	0.2595	0.2635	-0.0040	4.0000	0.000000	0.000000
1.38	3.7261	3.7163	0.0098	0.2739	0.2837	-0.0098	4.0000	0.000000	0.000000
1.43	3.7295	3.7154	0.0141	0.2705	0.2846	-0.0141	4.0000	0.000000	0.000000
1.48	3.7607	3.7443	0.0163	0.2393	0.2557	-0.0163	4.0000	0.000000	0.000000
1.53	3.8266	3.8125	0.0141	0.1734	0.1875	-0.0141	4.0000	0.000000	0.000000
1.58	3.9057	3.8987	0.0070	0.0943	0.1013	-0.0070	4.0000	0.000000	0.000000
1.63	3.9569	3.9548	0.0021	0.0431	0.0452	-0.0021	4.0000	0.000000	0.000000

Table S6: Orbital occupation number and spin numbers of Li_4 with CAS(4,4)-CCSD/QCC.

bond length	$Ne^{\mathrm{HOMO}}_{\mathrm{CCSD}}$	$Ne^{\mathrm{HOMO}}_{\mathrm{QCC}}$	$\Delta Ne^{\mathrm{HOMO}}$	$Ne^{\mathrm{LUMO}}_{\mathrm{CCSD}}$	$Ne^{\mathrm{LUMO}}_{\mathrm{QCC}}$	$\Delta Ne^{\mathrm{LUMO}}$	$Ne^{\mathrm{Total}}_{\mathrm{QCC}}$	S_z	S^2
2.46	3.9823	3.9817	0.0005	0.0177	0.0183	-0.0005	4.0000	0.000000	0.000000
2.56	3.9806	3.9801	0.0006	0.0194	0.0199	-0.0006	4.0000	0.000000	0.000000
2.66	3.9789	3.9783	0.0006	0.0211	0.0217	-0.0006	4.0000	0.000000	0.000000
2.76	3.9771	3.9765	0.0007	0.0229	0.0235	-0.0007	4.0000	0.000000	0.000000
2.86	3.9753	3.9746	0.0007	0.0247	0.0254	-0.0007	4.0000	0.000000	0.000000
2.96	3.9734	3.9726	0.0008	0.0266	0.0274	-0.0008	4.0000	0.000000	0.000000
3.06	3.9714	3.9705	0.0009	0.0286	0.0295	-0.0009	4.0000	0.000000	0.000000
3.16	3.9693	3.9683	0.0010	0.0307	0.0317	-0.0010	4.0000	0.000000	0.000000
3.26	3.9671	3.9662	0.0010	0.0329	0.0338	-0.0010	4.0000	0.000000	0.000000
3.36	3.9649	3.9638	0.0011	0.0351	0.0362	-0.0011	4.0000	0.000000	0.000000

Table S7: Orbital occupation number and spin numbers of O_3 with CAS(6,6)-CCSD/QCC.

bond length	$Ne_{\mathrm{CCSD}}^{\mathrm{HOMO}}$	$Ne_{\mathrm{QCC}}^{\mathrm{HOMO}}$	$\Delta Ne^{\mathrm{HOMO}}$	$Ne_{\mathrm{CCSD}}^{\mathrm{LUMO}}$	$Ne_{\mathrm{QCC}}^{\mathrm{LUMO}}$	$\Delta Ne^{\mathrm{LUMO}}$	$Ne_{\mathrm{QCC}}^{\mathrm{Total}}$	S_z	S^2
1.18	5.8354	5.8349	0.0005	0.1646	0.1651	-0.0005	6.0000	0.000011	0.000011
1.23	5.8059	5.8049	0.0010	0.1941	0.1951	-0.0010	6.0000	0.000015	0.000015
1.28	5.7771	5.7767	0.0004	0.2229	0.2233	-0.0004	6.0000	0.000005	0.000005
1.33	5.7514	5.7449	0.0066	0.2486	0.2551	-0.0066	6.0000	0.000008	0.000008
1.38	5.7324	5.7309	0.0014	0.2676	0.2691	-0.0014	6.0000	0.000011	0.000011
1.43	5.7254	5.7256	-0.0002	0.2746	0.2744	0.0002	6.0000	0.000011	0.000011
1.48	5.7378	5.7510	-0.0132	0.2622	0.2490	0.0132	5.9999	0.000016	0.000016
1.53	5.7747	5.8037	-0.0290	0.2253	0.1963	0.0290	6.0000	0.000009	0.000009
1.58	5.8249	5.8650	-0.0401	0.1751	0.1351	0.0400	6.0001	0.000051	0.000051
1.63	5.8641	5.9081	-0.0440	0.1359	0.0918	0.0441	5.9999	0.000025	0.000025

Table S8: Orbital occupation number and spin numbers of Li_4 with CAS(6,6)-CCSD/QCC.

bond length	$Ne_{\mathrm{CCSD}}^{\mathrm{HOMO}}$	$Ne_{\mathrm{QCC}}^{\mathrm{HOMO}}$	$\Delta Ne^{\mathrm{HOMO}}$	$Ne_{\mathrm{CCSD}}^{\mathrm{LUMO}}$	$Ne_{\mathrm{QCC}}^{\mathrm{LUMO}}$	$\Delta Ne^{\mathrm{LUMO}}$	$Ne_{\mathrm{QCC}}^{\mathrm{Total}}$	S_z	S^2
2.46	5.9717	5.9708	0.0009	0.0283	0.0292	-0.0009	6.0000	0.000000	0.000000
2.56	5.9702	5.9693	0.0009	0.0298	0.0307	-0.0009	6.0000	0.000000	0.000000
2.66	5.9686	5.9676	0.0010	0.0314	0.0324	-0.0010	6.0000	0.000000	0.000000
2.76	5.9669	5.9658	0.0011	0.0331	0.0342	-0.0011	6.0000	0.000000	0.000000
2.86	5.9652	5.9640	0.0012	0.0348	0.0360	-0.0012	6.0000	0.000000	0.000000
2.96	5.9633	5.9621	0.0012	0.0367	0.0379	-0.0012	6.0000	0.000000	0.000000
3.06	5.9614	5.9601	0.0013	0.0386	0.0399	-0.0013	6.0000	0.000000	0.000000
3.16	5.9594	5.9580	0.0014	0.0406	0.0420	-0.0014	6.0000	0.000000	0.000000
3.26	5.9573	5.9559	0.0014	0.0427	0.0441	-0.0014	6.0000	0.000000	0.000000
3.36	5.9552	5.9537	0.0014	0.0448	0.0463	-0.0015	6.0000	0.000000	0.000000
